# Supplementary figures and images for: Modulation of Brain Activity by Selective Attention to Audiovisual Dialogues
Source: Front Neurosci. 2020 May 12;14:436. doi: 10.3389/fnins.2020.00436 (PMC7235384; doi:10.3389/fnins.2020.00436)

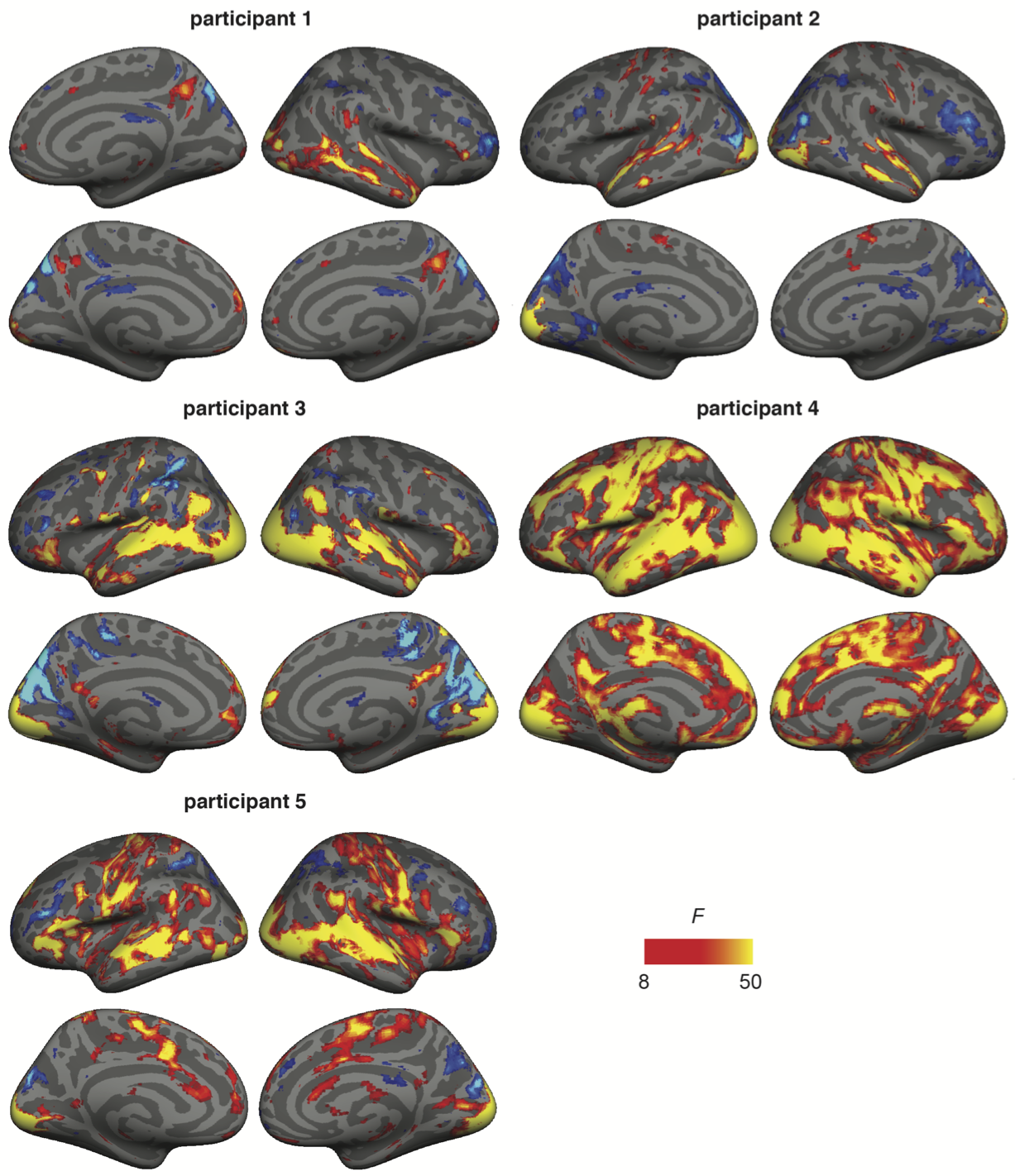

Supplement: FIGURE S1 — Correlations of the behavioral data with BOLD signal in selected ROIs. Y-axis: behavioral quiz scores (1–7), x-axis: BOLD signal change (%). A1–A3 denote poor, medium and good auditory quality; V1–V3 denote poor, medium and good visual quality. Each experimental condition is depicted in different color. STG, superior temporal gyrus (a, m, and p for anterior, mid and posterior, respectively); STS, superior temporal sulcus; HG, Heschl’s gyrus; FFA, fusiform face area. [file Image_1.TIFF]

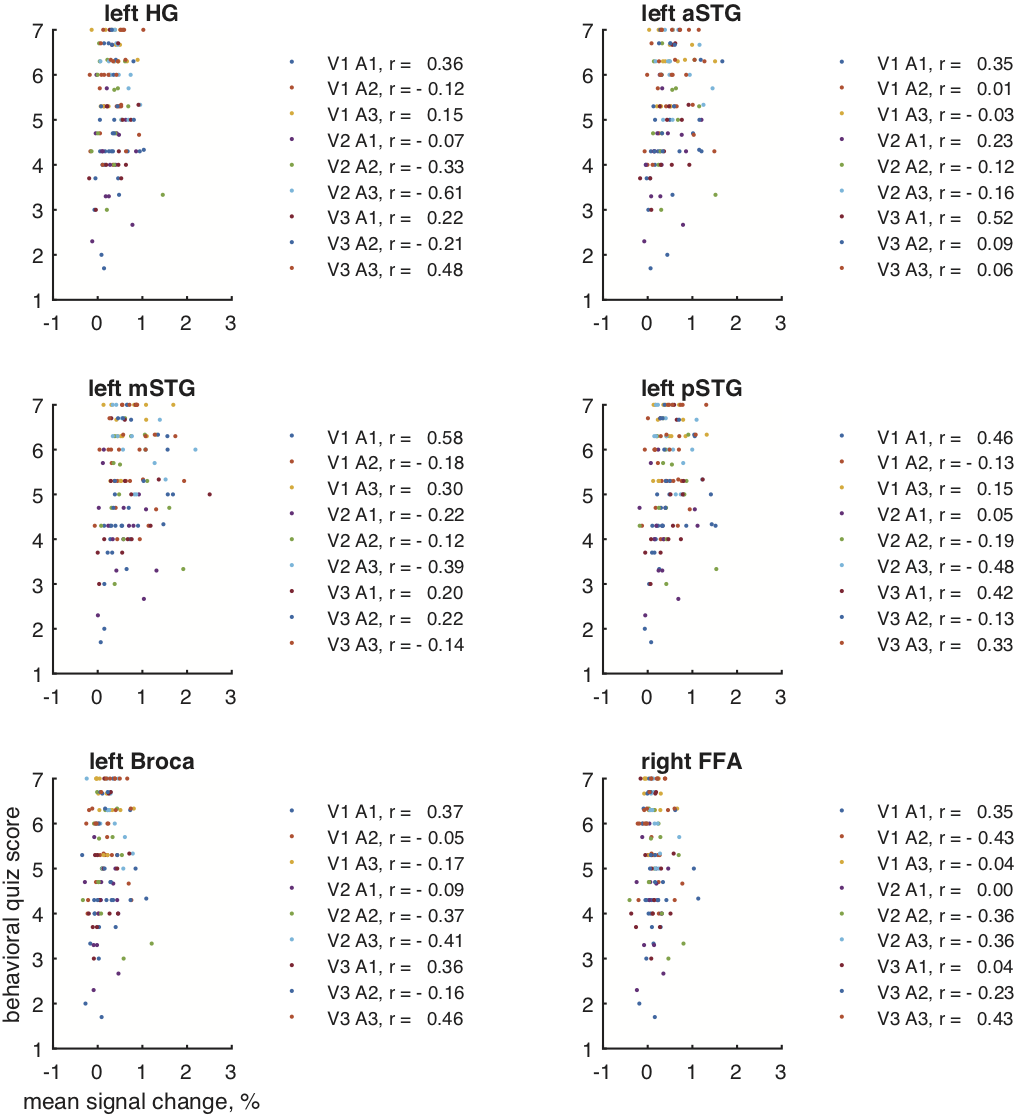

Supplement: FIGURE S2 — Raw BOLD activity (first run) for five participants in selected ROIs. Y-axis: time, volume, x-axis: mean BOLD, arbitrary unit. STG, superior temporal gyrus (a, m, and p for anterior, mid and posterior, respectively); STS, superior temporal sulcus; HG, Heschl’s gyrus; FFA, fusiform face area. [file Image_2.TIFF]

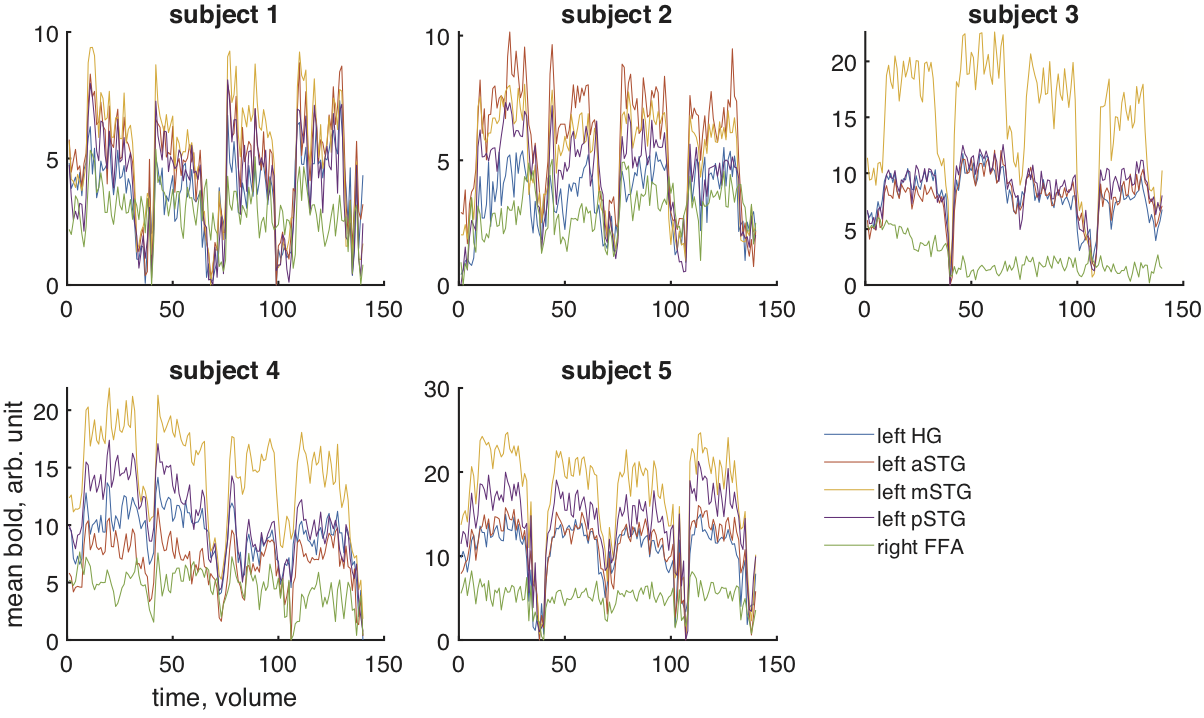

Supplement: FIGURE S3 — The average signal changes across all conditions. [file Image_3.TIFF]

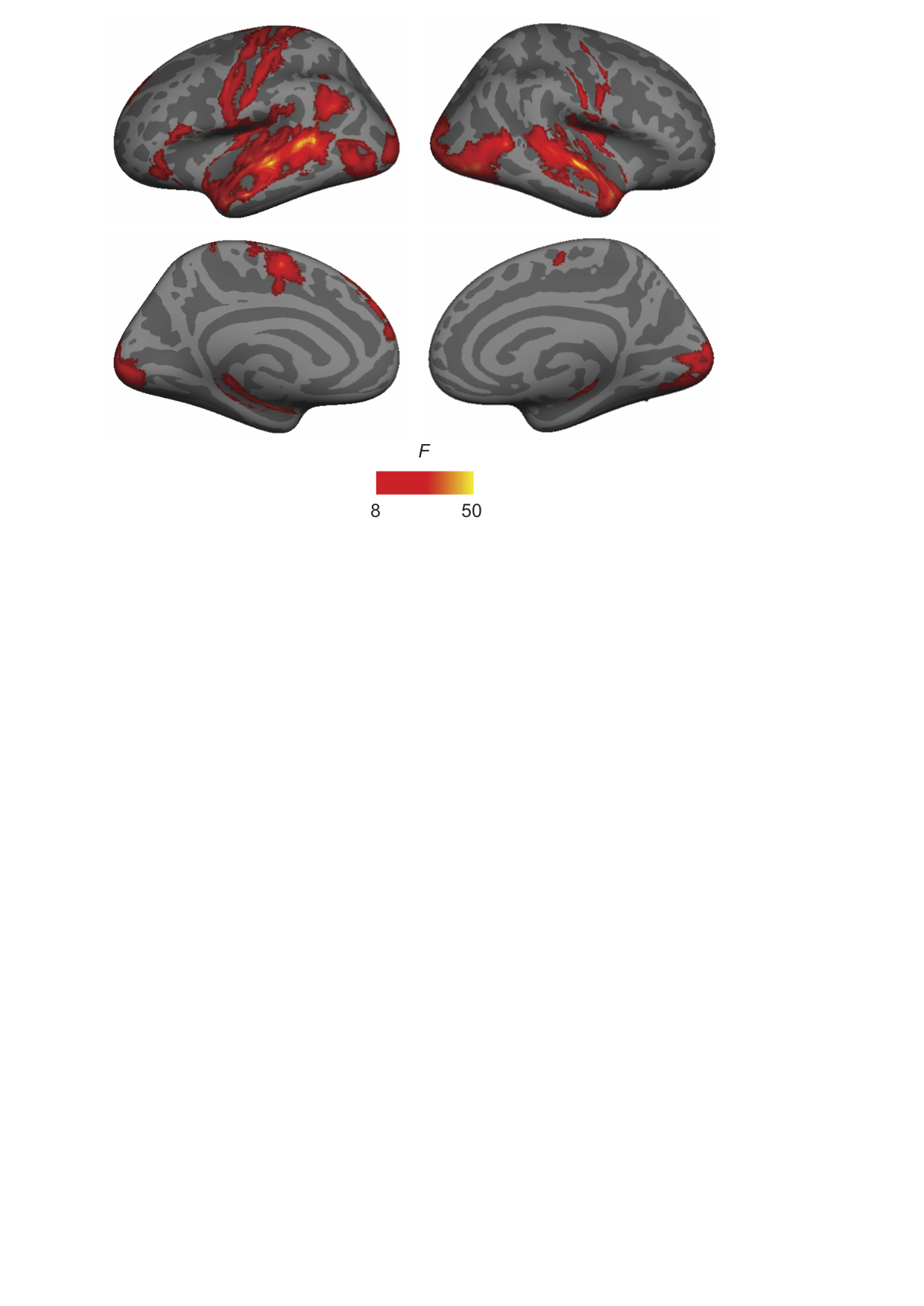

Supplement: FIGURE S4 — Individual participant data for 5 participants across all conditions. [file Image_4.TIFF]
